# Structure Determination of Novel Oxidation Products from Epicatechin: Thearubigin-Like Molecules

**DOI:** 10.3390/molecules21030273

**Published:** 2016-02-26

**Authors:** Kazuhiro Uchida, Kazuki Ogawa, Emiko Yanase

**Affiliations:** Faculty of Applied Biological Sciences, Gifu University, 1-1 Yanagido, Gifu 501-1193, Japan; u8121006@edu.gifu-u.ac.jp (K.U.); t8121010@edu.gifu-u.ac.jp (K.O.)

**Keywords:** epicatechin, oxidation, thearubigin, theaflavine, polymeric polyphenol, black tea

## Abstract

Following the oxidation of epicatechin (EC), three novel compounds and two known compounds were isolated. The chemical structures of these oxidation products were determined by mass spectrometry (MS) and various nuclear magnetic resonance (NMR) experiments, and the A-ring–B-ring linkage that is characteristic of catechin was found in each molecule. Three compounds showed similar ultraviolet–visible (UV-Vis) spectra to EC, whereas two compounds showed different spectral absorption in the region between 300 and 500 nm. A similar spectrum was obtained for the thearubigin fraction prepared from a black tea infusion. This result suggests that the condensation reaction between the A-ring and B-ring is more important than reaction between B-rings for thearubigin formation.

## 1. Introduction

Black tea is a globally popular beverage that has attracted attention because of its health benefits, including antioxidative and anticarcinogenic activities. Tea can be classified by differences in processing methods, for example, black tea is a fermented tea. The term “fermented” refers to natural browning reactions induced by oxidative enzymes of the fresh tea leaves. Catechins are one of the components in tea that are drastically changed in this process, and dimers and polymers are generated. The polymer found in black tea is called thearubigin. The name thearubigin was given to an ill-defined group of substances by Roberts in 1962. Thearubigin has a rust-brown color and high water solubility. Many researchers have studied the chemical structure of thearubigin, and the average molecular weight was estimated to be approximately 5000 to 30,000. Thearubigin constitutes approximately 10%–20% of the dry weight of black tea leaves; however, detailed chemical studies have thus far been limited because the numerous components found in the extract leads to a complicated mixture, which makes preparative scale isolation difficult [1,2,3,4].

Theaflavins [5,6,7,8] are one of the pigments in black tea that have a familiar bright red tone and various bioactivities [9,10]. In the synthesis of theaflavin from epicatechin (**1**, EC) and epigallocatechin, the synthetic yield increased when an excessive amount of **1** was used for this reaction. However, the recovery of **1** was low after the reaction. This result suggested that another reaction could occur between catechol-type catechins. We consider reactions between catechol-type catechins likely to occur during the fermentation process of black tea and thus be involved in thearubigin generation. Therefore, it is important to clarify the chemical structures of the byproducts from the synthesis of theaflavin.

In this study, to increase knowledge of the chemical structure of thearubigin, we investigated the oxidation reaction of **1**. Structure determination of the three novel and two known compounds isolated showed the importance of condensation reactions between catechin A- and B-rings for thearubigin formation.

## 2. Results and Discussion

**Figure 1:**
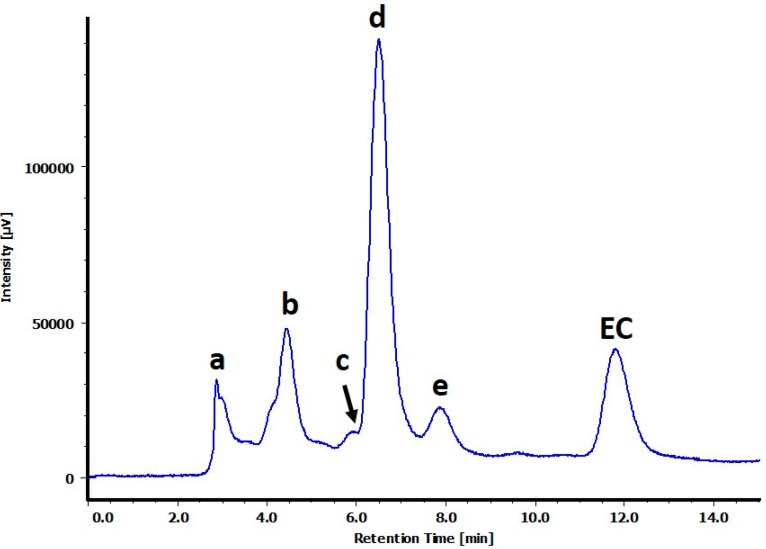
HPLC chromatogram of the reaction mixture obtained following oxidation of epicatechin (EC) (monitored at 280 nm). Peaks were shown as a–d. a: dehydrotricatechin B_2_ (**5**), b: dehydrotricatechin B_1_ (**6**), **c**: epicatechin-6′,6-dimer (**2**), d: epicatechin-6′,8-dimer (**3**), e: dehydrotricatechin A (**4**), EC: epicatechin (**1**).

Oxidation of **1** was performed using copper chloride, and the reaction mixture was analyzed using high-performance liquid chromatography with ultraviolet detection (HPLC-UV) (Figure 1). As a result, **1** and five unknown peaks, which were determined to be two dimers (peak c and d) and three trimers (peak a, b and e), were observed. The yield of each peak depended on the reaction conditions, oxidizing agent, reagent equivalents, reaction time, *etc.* For isolation and structure determination of each peak, the reaction was performed under optimum conditions for that peak. The reaction solution was extracted sequentially with ether and ethyl acetate. According to the HPLC analysis of each layer, the water layer contained peaks a and b, whereas the ethyl acetate layer contained peaks c, d, and e. Unreacted **1** was extracted into the ether layer. To collect peaks a and b, the water layer was loaded into a column packed with Diaion HP20SS particles and eluted with MeOH. Each peak was separated using preparative HPLC and characterized using spectroscopic techniques, including 1D and 2D nuclear magnetic resonance (NMR) and mass spectrometry.

### 2.1. Structure Determination for Peaks c and d

**Figure 2:**
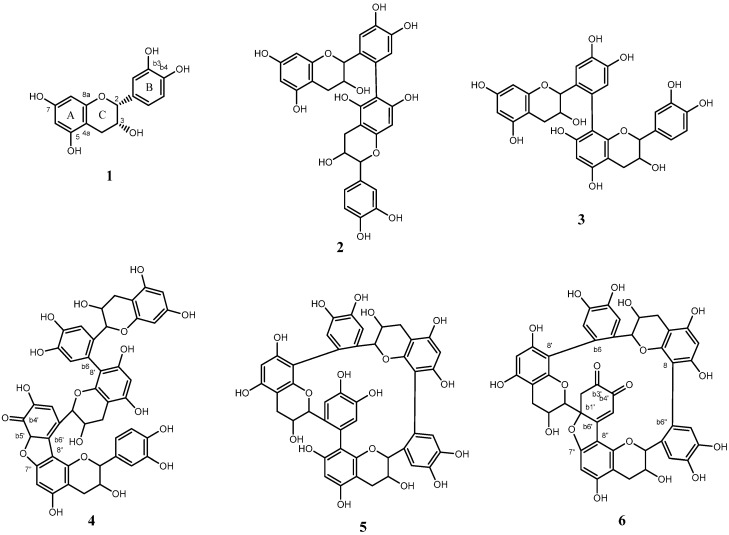
Chemical structures of compounds **1**–**6**.

Negative mode electrospray ionization mass spectrometry (ESIMS) measurements of peaks c and d both showed a pseudo-molecular ion peak at *m*/*z* 577 ([M − H]^−^), suggesting that these compounds are dimers of **1**. The ^1^H-NMR spectra of peaks **c** and **d** were similar and indicated the presence of two EC units that had lost one aromatic proton from both the A-ring and B-ring. Furthermore, the ^1^H- and ^13^C-NMR spectral signals were assigned using correlation spectroscopy (COSY), heteronuclear multiple quantum coherence (HMQC), and heteronuclear multiple-bond correlation (HMBC). As a result, the chemical structure of peak **c** was deduced to be the EC dimer with a connection between the 6-position of the A-ring in one molecule and the 2′-position of the B-ring in the other (compound **2**). On the other hand, peak d was found to be the EC dimer with a connection between the 8-position of the A-ring and the 2′-position of the B-ring (compound **3**), as shown in Figure 2. Compounds **2** and **3** have with been previously reported as the products of radical oxidation of **1**, and the spectral data were in agreement published data [11]. As a result, compounds **2** and **3** were identified as epicatechin-6′,6-dimer and epicatechin-6′,8-dimer.

### 2.2. Structure Determination for Peak e

**Figure 3:**
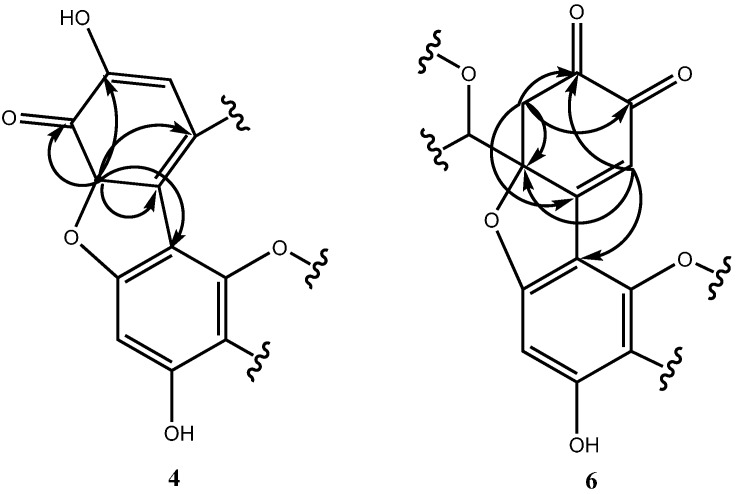
HMBC correlations for compounds **4** and **6**.

Negative mode ESIMS measurements of peak e showed a pseudo-molecular ion peak at *m*/*z* 863 ([M − H]^−^), suggesting that this compound is a trimer of **1**. The mass number was 2 Da smaller than the expected molecular weight calculated for a simple EC trimer with connections between EC units similar to those in dimers **2** and **3**. This result indicated that the EC units were connected via a double bond or three linkages. The ^1^H-NMR spectrum showed the presence of three pairs of signals corresponding to the 2-positions and 3-positions of the C-rings, indicating that these rings were unmodified. HMQC and HMBC data showed that the linkages of the three catechin units were between the A- and B-rings. In the ^13^C-NMR spectrum, a characteristic signal at 190 ppm indicated the presence of a ketone. This signal was correlated with methine (3.1 ppm), which, in HMBC, was correlated with the 7- and 8-positions of an A-ring and 1′-position of a B-ring, as shown in Figure 3. These results indicated the presence of a ketone in a B-ring that had lost aromaticity and the formation of a five-membered ring via an ether bond between the A-ring of one EC unit and the B-ring of another unit. Furthermore, the ^1^H- and ^13^C-NMR spectral signals were assigned based on COSY, HMQC, and HMBC (Figure 3). As a result, the structure of compound **4** was determined, as shown in Figure 2. We have named **4** dehydrotricatechin A.

### 2.3. Structure Determination for Peaks a and b

Peak a was analyzed by negative mode ESIMS. The appearance of an [M − H]^−^ ion peak at *m*/*z* 863, which was the same as that observed for compound **4**, suggested that compound **5** is a trimer of **1** with three linkages. In contrast to those of **4**, the ^1^H- and ^13^C-NMR spectra of **5** were simple and similar to those of monomeric **1**, except for the lack of one aromatic proton from each A-ring and B-ring. Although compound **5** was clearly different from **1**, we could not obtain further spectral evidence to confirm the complete structure of peak **a**.

Peak b showed a [M − H]^−^ peak at *m*/*z* 861 in negative mode ESIMS, indicating that this compound is another EC trimer. Three sets of signals arising from the 2-positions and 3-positions of the C-rings were observed in the ^1^H-NMR spectrum, indicating the presence of unmodified C-rings. The HMBC correlations observed between A-ring protons and B-ring carbons indicated that the EC units are connected between the A- and B-rings. The ¹³C-NMR signals at 190 and 180 ppm indicated the presence of two ketone groups. Based on HMQC, the correlations between these ketones and protons with signals at 4.47 and 2.81 ppm indicated that the ketones are geminal. Furthermore, the HMBC correlations of these ketones indicated a chemical skeleton that could be attributed to the 1,4-conjugated addition of the phenolic OH in the A-ring to *O*-quinone, resulting in a loss of aromaticity. All remaining signals in the ^1^H- and ^13^C-NMR spectra were assigned based on COSY, HMQC, and HMBC (Figure 3). As a result, the structure of compound **6** was determined, as shown in Figure 2. We have named **6** dehydrotricatechin B_1_.

Compound **6** was unstable in CD_3_OD and was converted to another compound with an NMR spectrum consistent with that of peak **a**. On the other hand, the CD_3_OD solution of peak **a** did not show conversion to compound **6**. This result indicated that compound **6** was irreversibly converted to compound **5**. Based on this observation and the NMR and ESIMS data described above, the chemical structure of compound **5** was proposed, as shown in Figure 2. We have named **5** dehydrotricatechin B_2_.

### 2.4. Formation Mechanisms of Compounds ***2**–**6***

**Figure 4:**
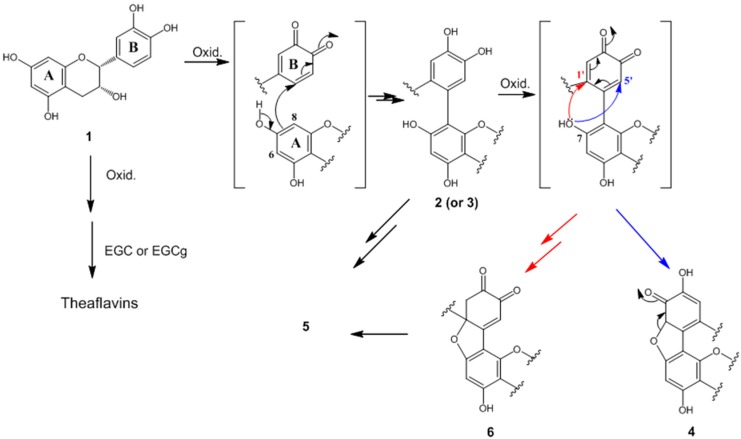
Formation mechanisms of compounds **2**–**6** from **1**.

On the basis of the chemical structures of compounds **2**–**6**, we proposed mechanisms for the formation of these compounds from **1**, as shown in Figure 4. When **1** is treated with an oxidizing agent, the catechol-type B-ring is oxidized to form *O*-quinone. The nucleophilic addition of the B-rings of epigallocatechin and epigallocatechin gallate to *O*-quinone occurs because of their high electron density, and eventually leads to the formation of theaflavins [11]. On the other hand, the nucleophilic addition of **1** to *O*-quinone occurs at the A-ring because of its higher electron density compared with that of the B-ring. Nucleophilic addition at the C8 or C6 position results in the formation of dimers, such as compounds **2** and **3**, or trimers, such as compound **5**. Furthermore, the catechol unit of the resulting product can be oxidized again to *O*-quinone and converted to compound **4** by intramolecular cyclization of the C7-hydroxy group with the C5′ position. Intramolecular cyclization can also occur with the C1 position to generate compound **6**. Compound **4** is converted into compound **5** through a ring opening reaction caused by aromatization. This formation mechanism suggested that the polymerization of catechin results from its oxidation, and further oxidation of these products results in the formations of compounds such as **4** and **6**.

### 2.5. UV-Vis Spectra

**Figure 5:**
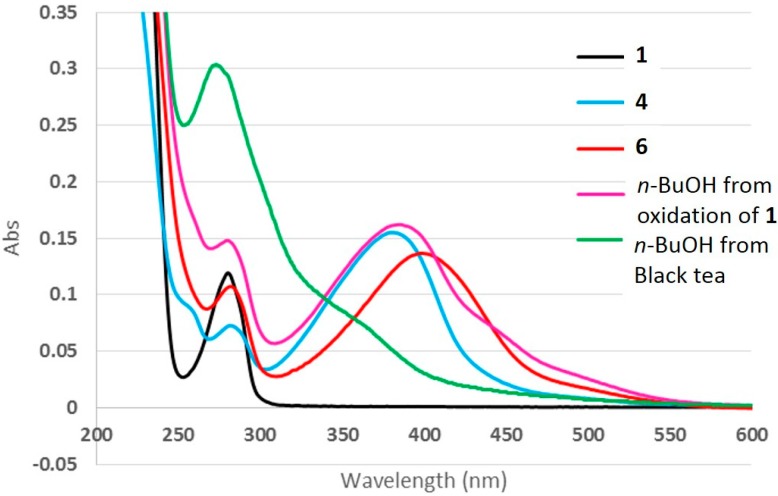
UV-Vis spectra of compounds **1**, **4**, and **6**, and the *n*-BuOH extracts.

To study the relationship between chemical structure and color, the ultraviolet–visible (UV-Vis) spectra of compounds **2**–**6** were obtained. Compounds **2**, **3**, and **5** showed similar spectra to that of **1**, which has a maximum absorption wavelength at 278 nm. On the other hand, compounds **4** and **6** showed spectral absorption in the region between 300 and 500 nm, which was not observed in the spectrum of **1** (Figure 5). This result indicated that the formation of the new five-membered ring in compounds **4** and **6** allowed conjugation between an A-ring and B-ring, resulting in absorption in the visible range.

Oxidation of **1** was performed under strong oxidation conditions using K_3_Fe(CN)_6_/NaHCO_3_, and the reaction mixture was extracted using ether, EtOAc, and *n*-BuOH, in the listed order. When each extract was analyzed by HPLC, the *n*-BuOH extract produced broad overlapping peaks. In addition, line broadening was observed in the ¹H-NMR spectrum of the *n*-BuOH extract. These results suggested that the *n*-BuOH extract contained thearubigin-like polymers that were generated by the oxidation reaction. The UV-Vis spectrum of this *n*-BuOH fraction showed similar spectral features to those of compounds **4** and **6**. This result indicated that the components in the *n*-BuOH fraction have chromophores with structures similar to those of compounds **4** and **6**.

Thearubigins are the most abundant pigments in black tea and are formed by enzymatic oxidization of catechins during the fermentation process. These compounds are polymeric polyphenols with unresolved structures [2,3,4]. To obtain the UV-Vis spectrum of these compounds, the hot water extract of black tea leaves was extracted using ether, EtOAc, and *n*-BuOH, in the listed order, and the *n*-BuOH extract was further fractionated into two fractions (Fr. 1 and 2) using a Toyopearl HW-40C column. The UV-Vis spectrum of Fr. 2, which had a reddish-brown color, showed a wide absorption band in the 300–500 nm region, similar to that of the *n*-BuOH extract of the oxidation mixture of **1** described above. The absorption band of the black tea extract was observed at 360 nm, which was somewhat blue-shifted in comparison to those of compounds **4** and **6** and the *n*-BuOH extract of the oxidation mixture of **1**. It is assumed that this difference is because thearubigins from black tea are a complex mixture that contains some products formed by conjugation between catechin and other components in addition to polymers derived only from catechins.

## 3. Experimental Section

### 3.1. Materials

EC was purchased from Sigma-Aldrich Co. LLC (St. Louis, MO, USA). Copper(II) chloride dihydrate and potassium hexacyanoferrate(III) were purchased from Nacalai Tesque Co. (Kyoto, Japan). Sodium hydrogen carbonate was purchased from Kishida Chemical Co., Ltd. (Osaka, Japan). CD_3_OD was purchased from Kanto Chemical Co. (Tokyo, Japan). Acetone-*d*_6_ was purchased from Acros Organics Co. (Morris Plains, NJ, USA).

### 3.2. Instrumentation

^1^H- and ^13^C-NMR, ^1^H-COSY, HMQC, and HMBC spectra were recorded on a JEOL ECA 600 spectrometer (JEOL, Tokyo, Japan) at 600 MHz for ^1^H and a Bruker Biospin AVANCE III 800 spectrometer (Bruker, Germany) at 200 MHz for ^13^C. ESIMS spectra were measured on a JMS-T100TD mass spectrometer (JEOL). Column chromatography was carried out using Diaion HP20SS (Mitsubishi Chemical Co., Tokyo, Japan) and Toyopearl HW-40C (Tosho Co., Tokyo, Japan). HPLC was performed with a JASCO PU-2089 or JASCO PU-887 system using a reverse-phase HPLC column (NB-ODS-9 4.6 mm I.D. × 250 mm, NX-ODS-9 10 mm I.D. × 250 mm, Nagara Science Co., Gifu, Japan) and JASCO MD-2010 or JASCO 875-UV detectors (JASCO Co., Tokyo, Japan). UV-Vis spectra were recorded on a Perkin Elmer Lambda 950 UV/VIS/NIR spectrometer (Kanagawa, Japan).

### 3.3. Synthesis of ***a*** and ***b***

An aqueous solution of EC (0.2 mmol) and CuCl_2_·2H_2_O (1.0 mmol) in H_2_O was vigorously stirred at room temperature for 3 h. The reaction mixture was extracted with Et_2_O. The water layer was extracted with EtOAc. The water layer was concentrated until EtOAc had been removed and the resulting solution was subjected to column chromatography using a HP20SS resin. The column was eluted with MeOH after washing with water to remove CuCl_2_, and the resulting solution was separated by reverse-phase HPLC (25% MeOH, 0.5% HCOOH in H_2_O) to afford **a** and **b**.

Compound **a** (**5**): ESIMS, *m*/*z*: 863 ([M − H]^−^). ^1^H-NMR (600 MHz, CD_3_OD) δ: 6.74 (1H, s, H-b2), 6.64 (1H, s, H-b5), 6.01 (1H, s, H-6), 4.41 (1H, s, H-2), 3.90 (1H, m, H-3), 2.56 (1H, dd, *J* = 16.5 and 4.1 Hz, H-4). ^13^C-NMR (200 MHz, CD_3_OD) δ: 155.6 (C-5), 153.3 (C-7), 152.7 (C-8a), 143.7 (C-b3), 143.2 (C-b4), 130.7 (C-b1), 123.6 (C-b6), 117.8 (C-b5), 115.0 (C-b2), 106.5 (C-8), 98.2 (C-4a), 94.2 (C-6), 76.2 (C-2), 63.8 (C-3), 27.3 (C-4).

Compound **b** (**6**): ESIMS, *m*/*z*: 861 ([M − H]^−^). ^1^H-NMR (600 MHz, acetone-d_6_) δ: 7.90 (1H, s, H-b2′′), 6.81 (1H, s, H-b5′′), 6.80 (1H, s, H-b5), 6.27 (1H, s, H-b2), 6.14 (1H, s, H-b5′), 6.10 (1H, s, H-6′), 6.07 (1H, s, H-6′′), 6.02 (1H, s, H-6), 4.73 (1H, s, H-2′′), 4.56 (1H, brs, H-3′), 4.47 (1H, d, *J* = 14.4 Hz, H-b2′), 4.45 (1H, brs, H-3′′), 4.36 (1H, s, H-2), 3.91 (1H, s, H-3), 3.52 (1H, s, H-2′), 2.9 (1H, H-4′; this peak could not be observed in ^1^H-NMR due to overlap with the H_2_O signal), 2.84 (2H, m, H-b2′, 4′′), 2.68 (1H, dd, *J* = 12.6 and 3.0 Hz, H-4′′), 2.59 (1H, d, *J* = 12.6 Hz, H-4), 2.17 (1H, dd, *J* = 12.3 and 3.0 Hz, H-4), 2.1 (1H, H-4′, this peak could not be observed in ^1^H-NMR due to overlap with the acetone signal). ^13^C-NMR (200 MHz, acetone-*d*_6_) δ: 190.8 (C-b3′), 177.4 (C-b4′), 163.8 (C-7′′), 162.9 (C-8a′′), 159.7 (C-b6′), 156.1 (C-5′), 156.0 (C-5′′), 155.4 (C-5), 154.2 (C-7′), 153.9 (C-8a), 153.4 (C-7), 150.6 (C-8a′), 145.6 (C-b4), 144.0 (C-b4′′), 143.8 (C-b3), 131.4 (C-b1), 130.0 (C-b6), 127.3 (C-b1′′), 119.7 (C-b6′′), 119.5 (C-b2), 118.3 (C-b5′), 117.9 (C-b5′′), 116.3 (C-b2′′), 114.7 (C-b5), 107.1 (C-8), 106.9 (C-8′), 103.4 (C-8′′), 102.3 (C-4a′′), 99.0 (C-4a′), 98.9 (C-4a), 95.7 (C-6′), 94.0 (C-6), 90.6 (C-6′′), 90.0 (C-b1′), 77.2 (C-2′), 76.8 (C-2′′), 76.7 (C-2), 64.6 (C-3′′), 63.0 (C-3), 61.6 (C-3′), 47.2 (C-2′), 31.1 (C-4′′), 29.4 (C-4′), 27.7 (C-4).

### 3.4. Synthesis of ***c*** and ***d***

An aqueous solution of EC (0.2 mmol) and CuCl_2_·2H_2_O (0.4 mmol) in H_2_O was vigorously stirred at room temperature for 1 h. The reaction mixture was extracted with Et_2_O. The water layer was extracted with EtOAc. The EtOAc layer was concentrated, and then separated by reverse-phase HPLC (25% MeOH, 0.5% HCOOH in H_2_O) to afford **c** and **d**.

### 3.5. Synthesis of ***e***

A solution of EC and K_3_Fe(CN)_6_ in NaHCO_3_ aq. was vigorously stirred at 0 °C for 6 min. The mixture was sequentially extracted with Et_2_O, EtOAc, and *n*-BuOH, in the listed order. The EtOAc layer was concentrated, and then separated by reverse-phase HPLC (25% MeOH, 0.5% HCOOH in H_2_O) to afford **e**. The *n*-BuOH fraction was concentrated to afford a residue.

Compound **e** (**4**): ESIMS, *m*/*z*: 863 ([M − H]^−^). ^1^H-NMR (600 MHz, CD_3_OD) δ: 7.04 (1H, d, *J* = 1.4 Hz, H-b2′′), 6.92 (1H, s, H-b2), 6.90 (1H, d, *J* = 6.0 Hz, H-b6′′), 6.81 (1H, d, *J* = 8.2 Hz, H-b5′′), 6.41 (1H, s, H-b5), 6.15 (1H, s, H-b2′), 6.13 (1H, d, *J* = 2.1 Hz, H-8), 6.07 (1H, s, H-6′′), 6.03 (1H, s, H-6′), 5.95 (1H, d, *J* = 2.0 Hz, H-6), 5.24 (1H, s, H-2′′), 5.00 (1H, d, *J* = 8.2 Hz, H-3′), 4.38 (1H, s, H-2), 4.26 (1H, brs, H-3′′), 4.16 (1H, d, *J* = 2.7 Hz, H-2′), 3.71 (1H, brs, H-3), 3.26 (1H, dd, *J* = 12.6 and 3.0 Hz, H-4′′), 3.1 (2H, m, H-b5′, 4′), 2.84 (1H, d, *J* = 12.0 Hz, H-4′′), 2.60 (1H, d, *J* = 13.8 Hz, H-4′), 2.57 (1H, d, *J* = 12.6 Hz, H-4), 2.31 (1H, dd, *J* = 12.6 and 3.6 Hz, H-4). ^13^C-NMR (200 MHz, CD_3_OD) δ: 191.3 (C-b4′), 165.0 (C-b3′), 164.1 (C-7′′), 163.5 (C-8a′′), 156.7 (C-8a), 156.3 (C-5), 155.8 (C-7), 154.8 (C-5′, 5′′), 153.6 (C-7′), 151.2 (C-8a′), 144.4 (C-b4′′), 144.3 (C-b3′′), 143.2 (C-b4, C-b3), 130.8 (C-b1′′), 129.6 (C-b1), 122.1 (C-b6), 118.1 (C-b6′′), 117.7 (C-b5), 114.7 (C-b5′′), 114.1 (C-b2), 113.9 (C-b2′′), 108.5 (C-b2′), 107.3 (C-8′), 104.5 (C-8′′), 101.5 (C-4a′′), 98.7 (C-4a′), 98.6 (C-4a), 95.6 (C-6′), 95.2 (C-8), 94.6 (C-6), 93.8 (C-b6′), 90.7 (C-6′′), 89.9 (C-b1′), 78.7 (C-2′), 78.6 (C-2′′), 76.1 (C-2), 68.1 (C-3′), 65.4 (C-3′′), 64.1 (C-3), 39.0 (C-b5′), 27.7 (C-4), 27.5 (C-4′′), 23.0 (C-4′).

### 3.6. Extraction of Black Tea

Black tea leaves (1.8 g) were refluxed in water at 120 °C for 30 min. After cooling, the solution was extracted sequentially with Et_2_O, EtOAc, and *n*-BuOH, in the listed order. The *n*-BuOH extract was concentrated under reduced pressure to afford a residue. The *n*-BuOH extract was further fractionated using a Toyopearl HW-40C column (40% acetone, 8 M urea in 0.1 M HCl aq.) to afford Fr. 1, which had a light yellowish-brown color, and Fr. 2, which had a reddish-brown color.

### 3.7. UV-Vis Spectra

Each sample was dissolved in methanol at a concentration of 0.01 mg/mL. The spectrum of each sample was collected in the spectral range between 200 and 800 nm with a spectral resolution of 1 nm. The spectra were recorded against the corresponding solvent as a baseline.

## 4. Conclusions

In conclusion, we investigated the oxidation reaction of **1** to increase knowledge of the chemical structure and formation mechanism of thearubigin. We determined the chemical structures of three novel trimers (compounds **4**–**6**) and two known dimers (compounds **2** and **3**). Furthermore, the UV-Vis spectrum of the black tea extract indicated that the chemical structures of thearubigins contain skeletons that are cyclized after the formation of linkages between A- and B-rings, as observed in compounds **4** and **6**. Oxidative condensation between the B-rings of catechins has been considered the formation mechanism of thearubigins. However, this result suggests that the condensation reaction between A- and B-rings is another important mechanism for thearubigin formation. We consider this result important for resolving the chemical structures of thearubigins, and further studies are now in progress.

## Abbreviations

The following abbreviations are used in this manuscript:

### Abbreviations

EC: Epicatechin
HPLC: High-performance liquid chromatography
ESIMS: Electrospray ionization mass spectrometry
NMR: Nuclear magnetic resonance
COSY: Correlation spectroscopy
HMQC: Heteronuclear multiple quantum coherence
HMBC: Heteronuclear multiple-bond correlation

## References

[1] Roberts E.A.H., Geissman T.A. (1962). Economic importance of flavonoid substances: Tea fermentation. The Chemistry of Flavonoid Compounds.

[2] Haslam E. (2003). Thoughts on thearubigins. Phytochemistry.

[3] Tanaka T., Kouno I. (2003). Oxidation of tea catechins: Chemical structures and reaction mechanism. Food Sci. Technol. Res..

[4] Yassin G.H., Koek J.H., Kuhnert N. (2015). Model system-based mechanistic studies of black tea thearubigin formation. Food Chem..

[5] Takino Y., Imagawa H., Horikawa H., Tanaka A. (1964). Studies on the mechanism of the oxidation of tea leaf catechins: Part III. Formation of a reddish orange pigment and its spectral relationship to some benzotropolone derivatives. Agric. Biol. Chem..

[6] Brown A.G., Falshaw C.P., Haslam E., Holmes A., Ollis W.D. (1966). The constitution of theaflavin. Tetrahedron Lett..

[7] Collier P.D., Bryce T., Mallows R., Thomas P.E., Frost D.J., Korver O., Wilkins C.K. (1973). The theaflavins of black tea. Tetrahedron.

[8] Shiraki M., Hara Y., Osawa T., Kumon H., Nakayama T., Kawakishi S. (1994). Antioxidative and antimutagenic effects of theaflavins from black tea. Mutat. Res. Lett..

[9] Yanase E., Sawaki K., Nakatsuka S. (2005). The isolation of a bicyclo[3.2.1] intermediate during formation of benzotropolones, a common nucleus found in black tea pigments: Theaflavins. Synlett.

[10] Ikeda I., Yamahira T., Kato M., Ishikawa A. (2010). Black-tea polyphenols decrease micellar solubility of cholesterol *in vitro* and intestinal absorption of cholesterol in rats. J. Agric. Food Chem..

[11] Sang S., Cheng X., Stark R.E., Rosen R.T., Yang C.S., Ho C.T. (2002). Chemical studies on antioxidant mechanism of tea catechins: Analysis of radical reaction products of catechin and epicatechin with 2,2-diphenyl-1-picrylhydrazyl. Bioorg. Med. Chem..

